# Ectopic third molars in the sigmoid notch: etiology, diagnostic imaging and treatment options

**DOI:** 10.1186/s13005-016-0133-x

**Published:** 2016-12-06

**Authors:** Marcel Hanisch, Leopold F. Fröhlich, Johannes Kleinheinz

**Affiliations:** Department of Cranio-Maxillofacial Surgery, University Hospital Münster, Albert-Schweitzer-Campus 1, Gebäude W 30, Münster, D-48149 Germany

**Keywords:** Dentigerous cyst, Ectopic third molar, Ectopic tooth, Mandibular notch, Sigmoid notch

## Abstract

**Background:**

The etiology of ectopic third molars located in the sigmoid notch of the mandible is unclear. Only a few cases have been reported. The aim of this article is to discuss the etiology as well as treatment options and diagnostic imaging techniques.

**Methods:**

A PubMed and Medline search of the literature from 1965 to 2015 to ectopic third molars in the mandibular notch was performed. Furthermore, a clinical case provided by the authors is reported.

**Results:**

Among the eight reviewed cases, two male and six female patients were affected that ranged from 25 to 62 years of age (mean 48.4). Pain and swelling in the preauricular region or trismus but also the absence of symptoms was reported. Only in two of the summarized articles an extra-oral access for the removal of the tooth was used. The etiology seems to be individually different, however dentigerous cysts and chronic inflammation seem to play an important role in their appearance. While previous diagnostic reports described two-dimensional diagnostic imaging, currently the three-dimensional imaging is common for preoperative surgical planning with respect to removing ectopic molars.

**Conclusions:**

Ectopic third molars in the mandible are a rare condition. The etiology seems to be individually different. Nowadays, three-dimensional imaging is common for preoperative surgical planning.

## Background

Ectopic molars in the mandible are rare cases and the etiology of this condition is still unclear [1]. Ectopic third molars of the mandible have been described in the condylar region, the coronoid process, the ascending ramus and the sigmoid notch. A review by Wang et al. indicated only 13 reported cases in the literature depicting ectopic molars in the ramus region during a period of 30 years [2]. The surgical excision of third molars is one of the most common outpatient surgeries [3], whereas the removal of ectopic molars seem to be an unusual surgical intervention. Preoperative diagnosis is based on clinical findings and diagnostic X-ray examination [4]. In the present paper, we review the literature of all cases describing ectopic third molars found in the mandibular sigmoid notch region, which have been reported over a period of 50 years from 1965 to 2015. Subsequently, we add to this summary our own experience by presenting a new case with an ectopic third molar in the sigmoid notch.

## Methods

A clinical case provided by the authors is reported. Furthermore, a literature search in PubMed and Medline databases was achieved by using the following MeSH terms: “sigmoid notch” OR “mandibular notch” AND “ectopic tooth” OR “third molar”. Inclusion criteria were international cases of ectopic third molars in the sigmoid notch, which have been reported in English or native language from 1965 to 2015.

## Results

From 1965 to 2015 only eight cases with ectopic third molars that occurred in the sigmoid notch of the mandible have been reported. In addition to six case reports which were written in English language [5–10], two cases that were presented in native language by an Italian and a Japanese group [11, 12], respectively, were also included. Clinical and radiological features of these eight cases are summarized in Table 1.

**Table 1:** Clinical and radiological features of ectopic molars in the sigmoid notch reported from 1965 to 2015

Author	Gender	Age	Symptoms	Surgical access	Radiology
Traiger J. et al. 1965 [5]	female	47	firm, hard swelling of the side of the face	extraoral, general anesthetic	posteroanterior and lateral jaw projection; encircling radiolucency
Giardino et al. 1966 [11] (Article in Italian)	female	62	trismus, sporadic pain praeauricular	none	posteroranterior roentgenogram, lateral oblique radiograph; encircling radiolucency
Nishijima et al. 1976 [12] (Article in Japanese)	female	60	trismus, pain and swelling in preauricular region	extraoral, general anesthetic	posteroranterior roentgenogram, lateral oblique radiograph; encircling radiolucency
Granite EL et al. 1985 [6]	female	60	none	none	panoramic radiograph; area of sclerotic bone
Metha DS et al. 1986 [7]	male	25	slowly growing swelling since 2 years	intraoral, general anesthetic	lateral oblique radiograph; radiolucent lesion
Balan N. 1992 [8]	female	30	pain in preauricular region	not specified	lateral oblique radiograph
Fidink Y et al. 2015 [9]	male	45	none	intraoral, general anesthetic	CT, panoramic radiograph; radiolucent lesion
Adachi M. et al. 2015 [10]	female	58	discomfort in the left buccal mucosa	intraoral, general anesthetic	CT, panoramic radiograph; radiolucent lesion

### Gender and age prevalence

Six female patients and two male patients were diagnosed with ectopic molars in the sigmoid notch. The age ranged from 25 to 62, with an average age of 48.4 years.

### Clinical symptoms

As clinical symptoms the eight reported cases describes pain [8], swelling [7], trismus [5], discomfort of the mucosa [10] as well as combinations of these symptoms [11, 12] or no symptoms [6, 9]. The clinical features of the eight reported cases are summarized in Table 2.

**Table 2:** Clinical Symptoms described in eight reported cases

Clinical Symptoms described in the eight reported cases	
Symptom	Author
Firm hard swelling with complete trismus	Traiger J. et al. 1965 [5]
Trismus and sporadic pain preauricularly	Giardino et al. 1966 [11] (Article in Italian)
Trismus, pain and swelling in preauricular region	Nishijima et al. 1976 [12] (Article in Japanese)
No symptoms	Granite EL et al. 1985 [6]
Slowly growing swelling for two years	Metha DS et al. 1986 [7]
Pain in the preauricular region	Balan N. 1992 [8]
No symptoms	Fidink Y et al. 2015 [9]
Discomfort in the left buccal mucosa	Adachi M. et al. 2015 [10]

### Treatment

Treatment was described in all cases except one [8]. Granite et al. reported periodic radiographic examination [6], Giordano et al. indicated denied treatment by the patient [11] whereas three authors referred their patients to intraoral access and extraction of the ectopic molar under general anesthesia [7, 9, 10]. Only two cases described extra-oral surgical access for the extraction of the ectopic molar [5, 12]. In detail, submandibular access was selected in both reports.

### Association with cystic lesions

Cystic lesions were described in four cases [5, 7, 9, 12]. Giordano et al. described encircling radiolucency [11]. Adachi et al. also reported encircling radiolucency which was diagnosed pathologically as granulation tissue [10]. One report referred to an area of sclerotic bone surrounding the tooth [6] whereas Balan did not describe any cystic lesion or other abnormalities which could be detected in the radiologic image [8].

### Diagnostic imaging

Diagnostic imaging techniques reports from 1992 to 1965 described lateral oblique radiographs [7, 8, 11, 12], a panoramic radiograph [6], or posteroranterior and lateral jaw projection [5, 11, 12]. Diagnostic imaging by three-dimensional methods, in addition to a two-dimensional panoramic radiograph, was only reported by Fidink et al. and Adachi et al. in 2015 [9, 10].

### Case presentation

**Fig. 1:**
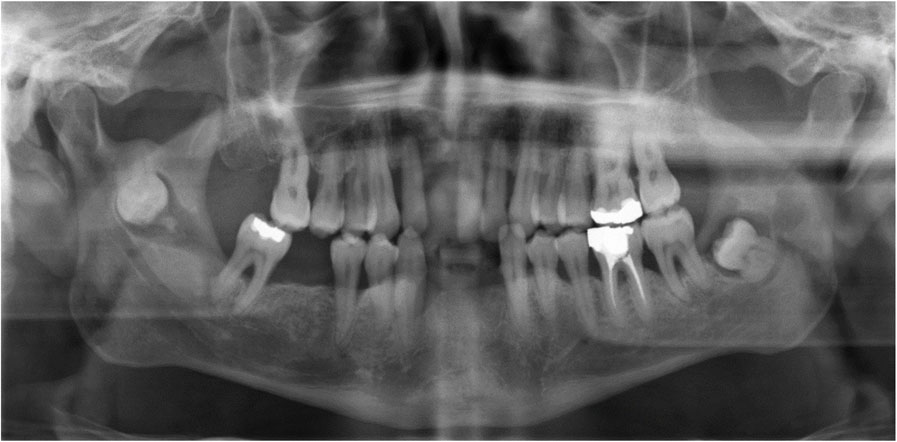
Panoramic radiograph showing the ectopic third right molar

**Fig. 2:**
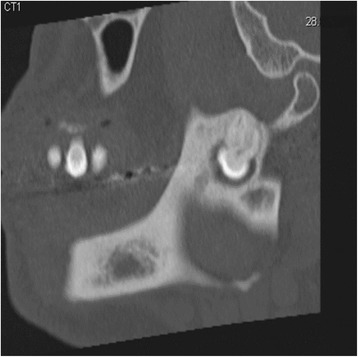
Sagital cone beam scan showing the impacted tooth with cranial-dorsal directed roots and bone apposition in the *right* sigmoid notch

**Fig. 3:**
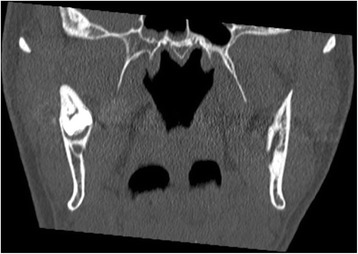
Coronal cone beam scan showing the impacted tooth with radiolucent cystic lesion superior the inferior alveolar nerve

**Fig. 4:**
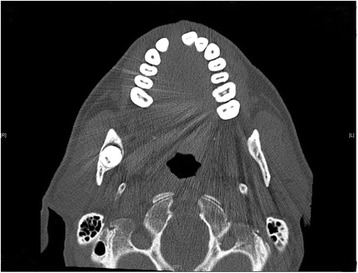
Axial cone beam scan showing the impacted tooth in the right sigmoid notch

**Fig. 5:**
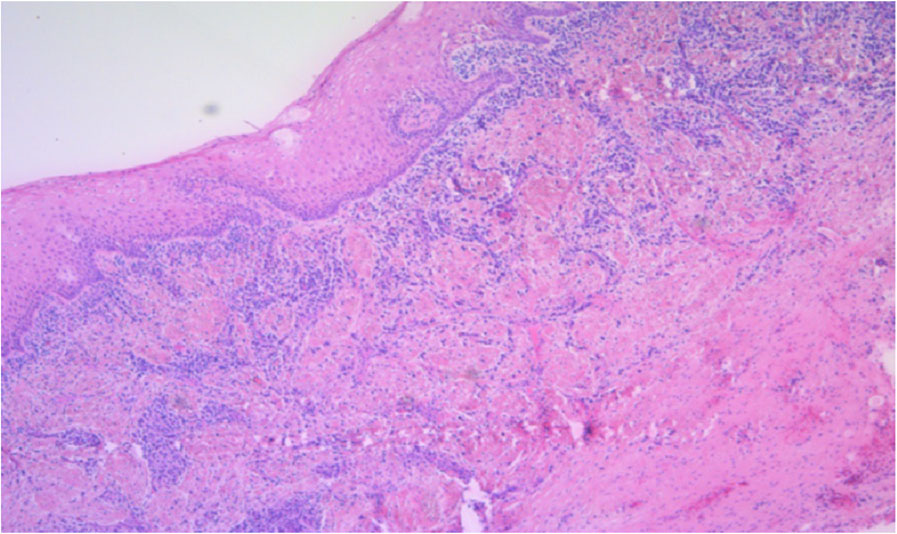
Microscopic image of the stratified epithelium demonstrating fibrous tissue with lymphocytic-, plasma cell- and neutrophilic granulocyte infiltration, as well as chronic inflammation (PAS, magnification: 100)

A 51 year-old male was referred to our Clinic of Cranio-Maxillofacial Surgery by his dentist. The patient described pain in the preauricular region for a few days. The panoramic radiograph revealed lower right third molar being dislocated in the sigmoid notch associated with a radioluscent lesion (Fig. 1). In addition, the panoramic radiograph offered generalized periodontitis and an impacted third molar surrounded with a radioluscent lesion on the left side of the mandible. Unfortunately, no earlier radiographic images of the patient were available for comparing the development of the ectopic molar. Clinical intra- and extraoral inspection disclosed no further inflammation signs like swelling, trismus, fever or redness. Also signs of chronic inflammation like fistula did not appear. Cone beam scans (CT) showed the impacted tooth with cranial-dorsal directed roots and bone apposition in the sigmoid notch (Figs. 2, 3, 4). A radiolucent cystic lesion was extending from the peri-coronary region of the tooth to the dental arch. The mandibular canal was compressed but covered by a small sclerotic bone (Fig. 1). Under endotracheal general anesthesia, an intraoral access was selected by incising the anterior edge of the mandibular ramus. In order to expose the sigmoid notch, a subperiosteal dissection was done lingually. Because the tooth was completely osseously covered, bone was removed and the tooth was separated with a surgical drill. The cystic lesion was enucleated and sent routinely for pathological analysis to the Department of Pathology, University Hospital Muenster. Microscopic analysis of the specimen showed stratified epithelium, fibrous tissue with lymphocytic-, plasma cell- and granulocytic infiltration of neutrophilic type and chronic inflammation (Fig. 5). Furthermore, all second molars and the third molar on the left mandible have also been removed. No complications occurred in the postoperative phase. Antibiotics were not given during the entire therapy. Subsequently, periodontal therapy will be performed by the patient’s dentist.

## Discussion

Up to now, only a few reports of ectopic third molars located in the mandible were recorded in the literature. The etiology of this condition is still unclear but several causes were discussed. Capelli described a correlation between the lack of space between second molar and the ramus mandibulae leading to an ectopic position of the impacted third molar [13]. Also a relationship involving the growth of the coronoid process and the ectopic position was suspected whenever the base of the ectopic third molar was embedded in the bony-growth tissue of the coronoid process [14]. Moreover, deviant eruption patterns were also assumed as a primordial deviance of the germ leading to ectopic teeth [15]. These theories may apply to be causative for the individual ectopic molars illustrated in the case reports which were summarized in this review. For the case presented in this article, the theory reported by Thoma in 1958 [16] and several other authors like Stafne [17] seems to apply for the identified ectopic molar. Thoma suspected that the pressure of the cystic fluid was responsible for the migration of the tooth. In our reported patient, a dentigerous cyst surrounds the crown. In the panoramic radiograph a radiolucent area similar to a “path” that extended from the dental arch to the ectopic molar in the sigmoid notch, appeared. Possibly, this “path” represents the route of migration starting at the dental arch and ending at the sigmoid notch. As inflammations are known to be supporting the expansion of cysts, the periodontitis determined in our patient could serve as an additional factor for the expansion of the cyst, leading to migration of the tooth. The same theory was reported by Adachi et al. which describes “granulation tissue with chronic inflammation around the crown” being etiological to the process of retrograde migration and forcing up the tooth into an ectopic position [10].

In symptomatic patients surgical removal, after a careful preoperative planning, is the recommended treatment [18]. In the past, diagnostic X-ray examinations were mainly implemented by two-dimensional diagnostic imaging techniques like panoramic radiograph or lateral jaw projection. Reports about complications during or after the removal of ectopic molars in the sigmoid notch like nerve injury, damage of the mandibular joint, bleeding or infections were not described in the reviewed literature. Ghaeminia et al. illustrated in their study that three-dimensional diagnostic imaging, compared to panoramic radiography, can contribute to optimal risk assessment and, as a consequence, allow better surgical planning [19]. Currently, three-dimensional diagnostic imaging techniques are established and can be beneficial in identifying position of the tooth, associated pathology and identifying the position of neurovascular structures [20]. Thus, preoperatively, the appropriate surgical method can be chosen [2].

## Conclusions

Ectopic third molars in the sigmoid notch of the mandible are a rare condition with higher prevalence in women. The etiology seems to be individually different, however dentigerous cysts and chronic inflammation seem to play an important role in their appearance. For planning the surgical entryway, which is mostly selected from intraoral as well as the assessment of operative-risks, three-dimensional diagnostic imaging techniques should be a preoperative standard in diagnostics.
